# Prp19 facilitates invasion of hepatocellular carcinoma via p38 mitogen-activated protein kinase/Twist1 pathway

**DOI:** 10.18632/oncotarget.7877

**Published:** 2016-03-03

**Authors:** Jie Yin, Lan Wang, Ji-Min Zhu, Qian Yu, Ru-Yi Xue, Ying Fang, Yi-An Zhang, Yan-Jie Chen, Tao-Tao Liu, Ling Dong, Xi-Zhong Shen

**Affiliations:** ^1^ Department of Gastroenterology, Zhongshan Hospital of Fudan University, Shanghai, China; ^2^ Department of Biochemistry and Molecular Biology, Shanghai Medical College of Fudan University, Shanghai, China; ^3^ Shanghai Institute of Liver Diseases, Zhongshan Hospital of Fudan University, Shanghai, China; ^4^ Key Laboratory of Medical Molecular Virology, Shanghai Medical College of Fudan University, Shanghai, China

**Keywords:** liver cancer, tumor progression, ubiquitination, signaling pathway

## Abstract

Pre-mRNA processing factor 19 (Prp19) is involved in many cellular events including pre-mRNA processing and DNA damage response. However, the pathological role of Prp19 in hepatocellular carcinoma (HCC) is still elusive. Here, we reported that Prp19 was increased in most HCC tissues and HCC cell lines, and its overexpression in HCC tissues was positively correlated with vascular invasion, tumor capsule breakthrough and poor prognosis. Prp19 potentiated migratory and invasive abilities of HCC cells *in vitro* and *in vivo*. Furthermore Prp19 facilitated Twist1-induced epithelial-mesenchymal transition. Mechanistic insights revealed that Prp19 directly binded with TGF-β-activated kinase1 (TAK1) and promoted the activation of p38 mitogen-activated protein kinase (MAPK), preventing Twist1 from degradation. Finally Prp19/p38 MAPK/Twist1 axis was attested in nude mice xenografts and HCC patient specimens. This work implies that the gain of Prp19 is a critical event during the progression of HCC, making it a promising target for malignancies with aberrant Prp19 expression.

## INTRODUCTION

Hepatocellular carcinoma (HCC), one of the most common malignancies, represents the third cause of cancer-related deaths worldwide [1]. Although screening in high-risk population has improved detection of early-stage HCC, most cases still present at advanced and unresectable stage because of intrahepatic metastasis and vascular invasion [2, 3]. The development of HCC is indeed a multistep process driving the progressive transformation of normal hepatocytes into highly malignant derivatives. Among them, epithelial-mesenchymal transition (EMT) is a key program that is often activated [4].

EMT, the initial step of tumor metastasis, is characterized by the loss of epithelial features and the acquisition of mesenchymal phenotypes [5]. This reversible cellular event is mediated by several critical transcription factors, including Snail 1/2, zinc-finger E-box binding homeobox 1/2 (ZEB1/2), forkhead box C2 and Twist1 [6]. Several lines of evidence have demonstrated that Twist1 is increased in HCC and its overexpression is positively correlated with invasiveness [7-9]. In spite of its significant role in invasiveness, mechanisms accounting for aberrant activity of Twist1 in HCC are poorly understood.

Ubiquitination is increasingly recognized to be important for the regulation of EMT [10]. During ubiquitination, deregulation of enzymes such as deubiquitinating enzyme (DUB) and ubiquitin-protein ligase (E3), has been linked to the initiation and the development of cancer [11, 12]. We previously demonstrated that ubiquitin carboxyl-terminal hydrolase 37 (UCH37), a member of the DUBs, promoted invasion and postoperative recurrence of HCC, and pre-mRNA processing factor 19 (Prp19) may function as its downstream effecter [13]. Prp19 itself possesses E3 activity and plays diverse roles in pre-mRNA processing and DNA damage response [14]. Despite disorders of these cellular events are linked to tumorgenesis, the potential role of Prp19 in HCC is unknown.

In this study, we demonstrate that Prp19 is overexpressed both in HCC patient specimens and tested HCC cell lines, and its elevation in HCC tissues positively correlates with vascular invasion, tumor capsule breakthrough and poor prognosis. Prp19 facilitates HCC invasion via Twist1-induced EMT. Moreover, Prp19 directly binds with TGF-β-activated kinase1 (TAK1) and promotes k-63 polyubiquitination of TAK1. This modulation leads to the activation of p38 mitogen-activated protein kinase (MAPK), suppressing proteasome-induced degradation of Twist1. These results presented here provide evidence for a novel function of Prp19 in HCC progression, which may rely on its non-proteolytic E3 activity.

## RESULTS

### Prp19 overexpression in HCC indicates invasiveness and poor prognosis

**Figure 1:**
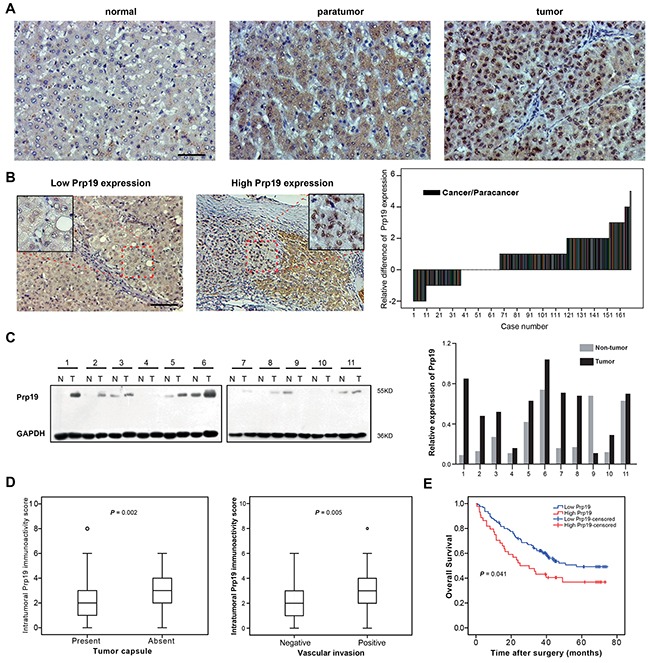
Prp19 overexpression in HCC indicates invasiveness and poor prognosis of HCC **A.** Representative images of IHC staining with Prp19 in liver tissues (from left to right: normal liver tissue, paratumor tissue and matched tumor tissue; Scale bar: 100μm). **B.** Representative view of Prp19 in HCC tumor tissues in IHC staining (Scale bar: 100μm). Micrograph indicated the amplified view of HCC tissue (original magnification ×400). Difference of Prp19 immunoactivity score in paired HCC tissue samples (N=169, right panel). **C.** Prp19 expression in tumor tissue (T) and paired paratumor tissue (N) from HCC patient specimens using western blot (left panel). Prp19 bands were quantified and shown in the bar chart after normalization (right panel). **D.** Relative immunoactivity score of Prp19 in HCC with or without tumor capsule (left panel) or vascular invasion (right panel). **E.** The overall survival rate between the low and high Prp19 expression groups in 169 HCC patients.

To investigate the role of Prp19 in liver tissues, IHC staining was performed with tissues from 13 normal liver and 169 HCC samples. Prp19 was mainly located at nucleus of HCC cells and its expression gradually increased from normal to paratumor and to paired tumor tissue of HCC (Figure 1A). In HCC tissues, Prp19 enrichment was observed at the edge of tumor and adjacent metastatic lesions (Supplementary Figure S1). In contrast to paired paratumor tissue, Prp19 was upregulated in most HCCs (102/169; Figure 1B), which was further confirmed in 11 cases by western blot (10/11) (Figure 1C).

Based on Prp19 expression in cancer tissues, all HCC patients were divided into high Prp19 expression group and low Prp19 expression group (detailed method for division listed in Supplementary Table S3). Relationship between Prp19 expression and clinicopathological features were presented in Supplementary Table S4. Prp19 overexpression was positively correlated with absent tumor capsule and vascular invasion (*P* = 0.002 and *P* = 0.005, respectively; Figure 1D). Further, patients in the high expression group exhibited shorter OS than those in the low expression group (median OS time, 25 and 57 months, respectively; *P* = 0.041; Figure 1E). These results demonstrate that enhanced Prp19 expression may act as a predicting factor for increased invasiveness and dismal prognosis in HCC patients.

### Prp19 enhances invasive potentials of HCC cells both *in vitro* and *in vivo*

In common with the findings in HCC patients, all tested HCC cell lines also displayed higher Prp19 protein level than that in normal hepatocyte L02 (Supplementary Figure S2A). In contrast to L02, only Hep3B and SMMC-7721 HCC cells showed evidently higher Prp19 mRNA expression (Supplementary Figure S2B).

**Figure 2:**
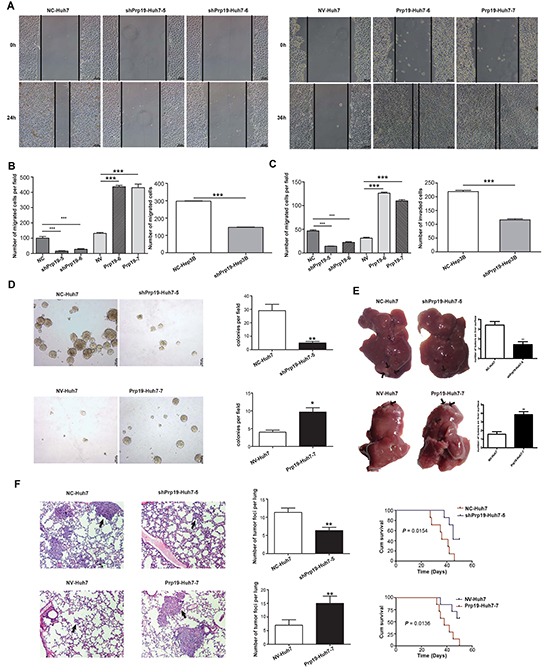
Prp19 enhances invasive potentials of HCC cells both *in vitro* and *in vivo* **A.** The migratory capacity of stable Huh7 cells mis-expressing Prp19 was analysed by wound-healing assay. **B.** Stable Huh7 and Hep3B cells mis-expressing Prp19 was subjected to transwell assay. **C.** The invasive capacity of Huh7 and Hep3B cells was analysed using transwell filter chambers coated with Matrigel. **D.** Representative images of colonies formed by stable Huh7 cells mis-expressing Prp19 in soft agar. **E.** Representative images of tumor in the liver of nude mice given orthotopic implantation of xenograft generated by stable Huh7 cells mis-expressing Prp19. Black arrows indicated metastatic lesions in liver. **F.** Representative images of hematoxylin & eosin staining of lung tissue sections derived from nude mice injected with stable Huh7 cells mis-expressing Prp19 in lateral tail vein. Black arrows indicated pulmonary metastasis (Original magnification ×100). The cumulative survival of nude mice in each group (n=7) was analysed by GraghPad Prism5. Migrated and invaded cells were plotted as the average number of cells per field of view from three different experiments. Error bar represents stand error of mean. *P < 0.05, **P < 0.01, ***P < 0.001. NC, negative control; NV, null vector.

To evaluate the effect of Prp19 on biological behaviours, stable HCC cells mis-expressing Prp19 were generated (Supplementary Figure S2C). Although previous study suggested that Prp19 displays proliferation-promoting effect on HeLa cells *in vitro* [17], Prp19 expression had marginal correlation with the proliferation of Huh7 cells (Supplementary Figure S3). Up-regulating Prp19 increased migratory capacity of Huh7 cells in cell migration and wound-healing assays (Figure 2A and 2B). Meanwhile, Prp19 down-regulation inhibited migratory capacity of Huh7 and Hep3B cells (Figure 2B). Matrigel invasion chamber assay revealed that Prp19 knockdown obviously inhibited invasiveness of Huh7 and Hep3B cells, whilst Prp19 overexpression significantly enhanced invasive potential of Huh7 cells in contrast to their controls (Figure 2C). Anchorage-independent growth is an important indicator to assess the invasive capacity of tumor cells *ex vivo*. In soft agar assay colonogenicity of Huh7 cells was decreased after repressing Prp19 expression; whilst Prp19-Huh7 cells formed more colonies than NV-Huh7 cells did (Figure 2D).

To verify the pro-invasive role of Prp19 *in vivo*, nude mice HCC model via orthotopic implantation of xenografts was generated. Less intrahepatic metastasis was induced by injecting with shPrp19-Huh7 cells than those injecting with NC-Huh7 cells, whereas more intrahepatic metastasis was generated by Prp19-Huh7 cells as compared with those generated by NV-Huh7 cells (Figure 2E). To further confirm the pro-invasive role of Prp19, stable Huh7 cells mis-expressing Prp19 were injected into lateral tail vein of 5-week-old nude mice. Four weeks later, less and smaller micrometastatic lesions were detected in the lungs of mice injected with shPrp19-Huh7 cells as compared with those injected with NC-Huh7 cells, whereas more and larger metastatic lesions were detected in the lungs of mice inoculated with Prp19-Huh7 cells as compared with those injected with NV-Huh7 cells. Moreover, mice injected with shPrp19-Huh7 cells had a longer survival period than those injected with NC-Huh7 cells, whilst mice inoculated with Prp19-Huh7 cells displayed a shorter survival period than those injected with NV-Huh7 cells (Figure 2F). These findings, together with observations *in vitro*, suggest that Prp19 promotes migration and invasion of HCC cells both *in vitro* and *in vivo*.

### Prp19 promotes Twist1-dependent EMT of HCC cells

**Figure 3:**
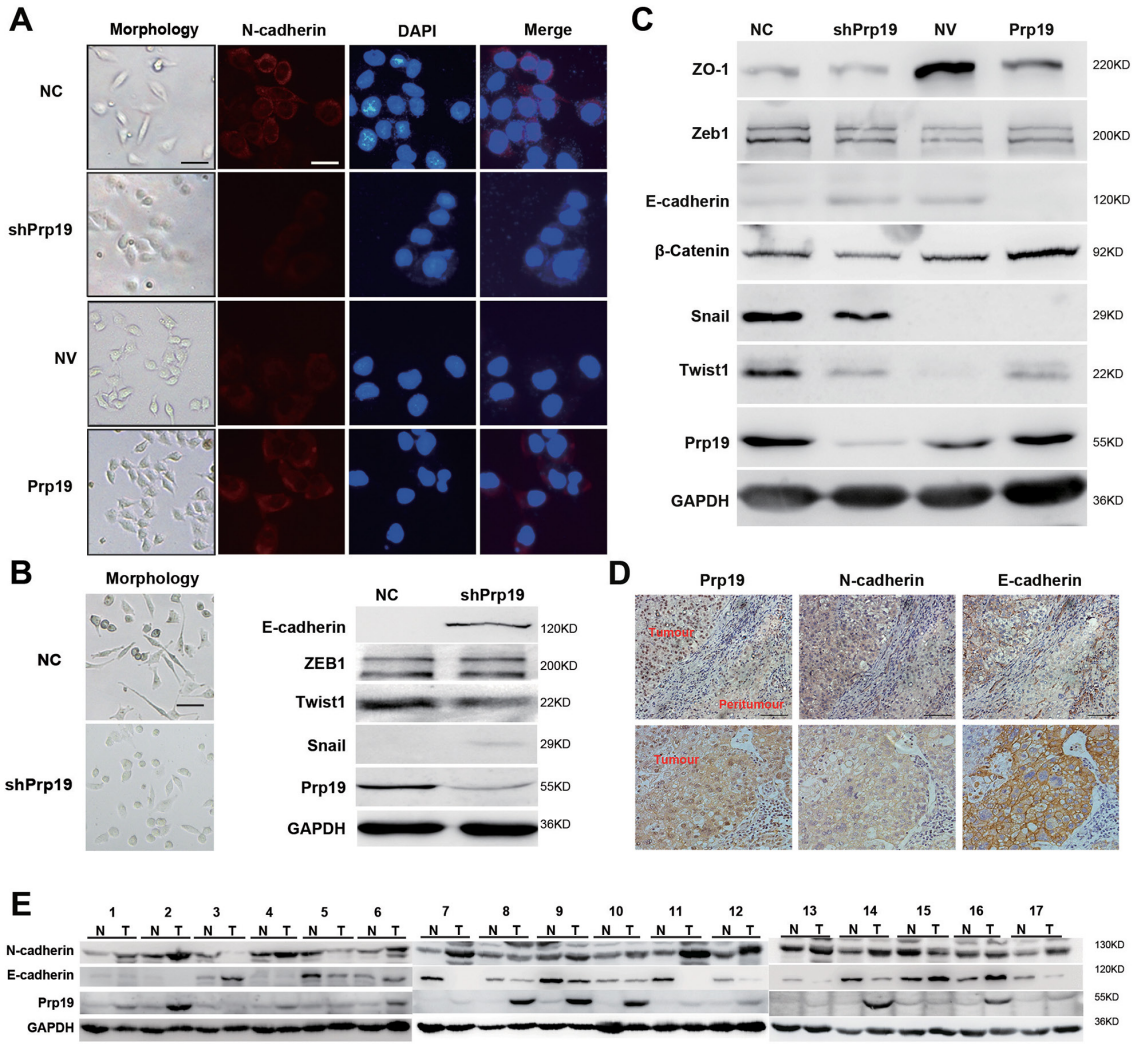
Prp19 promotes EMT of HCC cells **A.** Morphology of stable Huh7 cells mis-expressing Prp19 and respective N-cadherin expression via immunofluorescence assay (White scale bar: 50 μm; black scale bar: 100 μm). **B.** Morphology of NC-SK-Hep1 and shPrp19-SK-Hep1 cells (left panel). EMT biomarkers were analysed using western blot in NC-SK-Hep1 and shPrp19-SK-Hep1 cells (right panel). **C.** EMT biomarkers were analyzed using western blot in stable Huh7 cells mis-expressing Prp19. **D.** Representative images of IHC staining of Prp19, E-cadherin and N-cadherin in consecutive tissue sections from HCC (original magnification ×200). **E.** Western blot of Prp19, E-cadherin and N-cadherin in HCC tissues and paired paratumor tissues from 17 HCC patients.

Prp19 down-regulation in HCC cells gave rise to epithelial phenotypes with enhanced cell contact and cobblestone alteration, while Prp19 up-regulation displayed mesenchymal characteristics with decreased cell-cell adhesion and fibroblast-like appearance (Figure 3A and 3B, Supplementary Figure S4A). Prp19 knockdown exhibited the MET phenotypes (including decreased expression of β-catenin and N-cadherin, and increased expression of E-cadherin, while Prp19 up-regulation displayed the EMT phenotypes (increased expression of β-catenin and N-cadherin, and decreased expression of E-cadherin and ZO-1; Figure 3A, 3B, 3C and Supplementary Figure S4A). Alterations of E-cadherin and N-cadherin mRNA in stable Huh7 cells mis-expressing Prp19 was also detected (Supplementary Figure S4B). Similar correlation between the Prp19 level and the EMT markers was also observed in tested HCC specimens (Figure 3D and 3E). In the EMT transcription factors, Prp19 knockdown preferentially inhibited Twist1 expression in HCC cell lines (Figure 3C and 3D, Supplementary Figure S4A). RNAi silencing of Twist1 in Prp19-Huh7 cells [18], to some extent, changed its morphology from spindle shape with loose cell-cell contact to cubic shape with tight cell-cell contact (Supplementary Figure S5B). It also compromised the mobility and invasiveness of Prp19-Huh7 cells (Supplementary Figure S5C and S5D). These observations demonstrate that the pro-invasive role of Prp19 on HCC cells is mainly dependent on Twist1-induced EMT.

### Prp19 inhibits the ubiquitin/proteasome-dependent degradation of Twist1 in HCC cells

**Figure 4:**
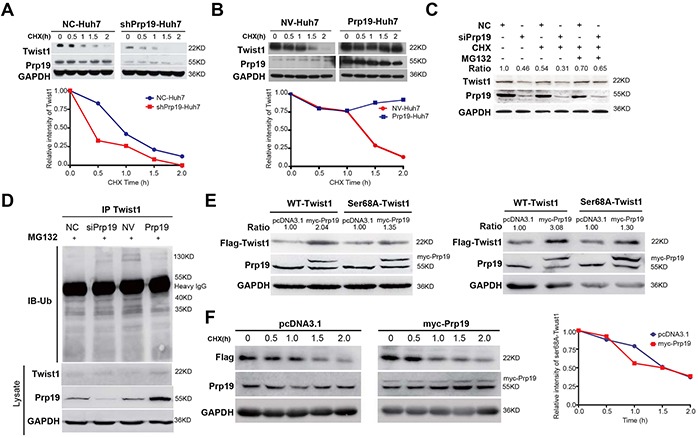
Prp19 inhibits the ubiquitin/proteasome-dependent degradation of Twist1 in HCC cells A, B.* Stable Huh7 cells mis-expressing Prp19 were treated with 100μg/ml cycloheximide (CHX) for indicated time, and then Twist1 expression were analysed. **C.** Huh7 cells were transfected with indicated siRNAs. After 48h transfection, Huh7 cells were treated sequentially with 50μg/ml CHX for 1h, 20μM MG132 for another 3h, and followed by western blot. **D.** Huh7 cells were transfected with indicated siRNAs or plasmids for 48h, and then treated with 20μM MG132 for another 6h. The ubiquitination of Twist1 was assessed by immunoprecipitation (IP) and immunoblot (IB). **E.** 293T cells (left panel) and Huh7 cells (right panel) were transfected with indicated plasmids, and then WT-Twist1 and Ser68A-Twist1 were detected using antibody against Flag. F.* Huh7 cells were transfected with indicated plasmids and treated with 100 μg/ml CHX for indicated time points, followed by western blot. *Relative densitometric values were detected and presented.

Although Prp19 is a pre-mRNA splicing factor, it had no effect on the mRNA expression of Twist1 in HCC cells (Supplementary Figure S6A). Since Twist1 is labile *in vivo* and the ubiquitin/proteasome pathway is responsible for Twist1 turnover [19], we next measured the stability of Twist1 in stable Huh7 cells mis-expressing Prp19. Cycloheximide half-life test demonstrated that Prp19 knockdown impaired Twist1 stability (Figure 4A), whilst Prp19 overexpression enhanced Twist1 stability (Figure 4B).

Three specific siRNAs against Prp19 were designed and siRNA3 displayed the most inhibitory effect and then was used in subsequent experiments (Supplementary Figure S6B). In contrast to Huh7 cells transfected with negative control siRNA, decrease of Twist1 induced by silencing Prp19 was reversed upon MG132 treatment (Figure 4C). Prp19 overexpression in Huh7 cells moderately decreased the amount of ubiquitinated protein in the Twist1 immuoprecipitates of Huh7 cells, whilst Prp19 downregulation increased the amount of ubiquitinated protein (Figure 4D). It is reported that ubiquitin/proteasome-dependent degradation of Twist1 is orchestrated by phosphorylation at residue serine (Ser) 68 or by dimerization formation with other transcription factors [20, 21]. No endogenous interaction between Prp19 and Twist1 was, however, found in Huh7 cells (Supplementary Figure S6C). In contrast to null vector, upregulating Prp19 in 293T cells, Huh7 and SK-Hep1 cells significantly increased WT-Twist1 level rather than Ser68A-Twist1 level (Figure 4E, Supplementary Figure S6D). Moreover, overexpressing Prp19 had no evident effect on Ser68A-Twist1 stability in Huh7 cells (Figure 4F). Moreover total Ser phosphorylation of Twist1 was positively correlated with Prp19 expression in HCC cells (Supplementary Figure S7A). Taken together, these results suggest that Prp19 represses ubiquitin/proteasome-dependent degradation of Twist1 by promoting its phosphorylation of Ser68 in HCC cells.

### Prp19 facilitates k63-linked polyubiquitination on TAK1 to activate p38 MAPK in HCC cells

Mitogen-activated protein kinase (MAPK) pathway is vital for Twist1 stability in breast cancer. Perturbation of MAPK pathway in Huh7 cells using specific inhibitors also displayed that inhibiting p38/MAPK activity significantly suppressed Twist1 expression (Supplementary Figure S7B), whilst activation of p38/MAPK using lipopolysaccharide (LPS) upregulated Twist1 expression, and this effect was reversed by SB203580 in a dose-dependent manner (Supplementary Figure S7C and S7D; using p-MAPKAPK2 as the indicator of p38/MAPK activity [22]). Repressing p38/MAPK activation also repressed total Ser phosphorylation of Twist1 (Supplementary Figure S7E, of note, there is no commercial antibody against phospho-Twist1 in Ser68). These results demonstrated that Twist1 was modulated by p38/MAPK pathway in HCC cells.

**Figure 5:**
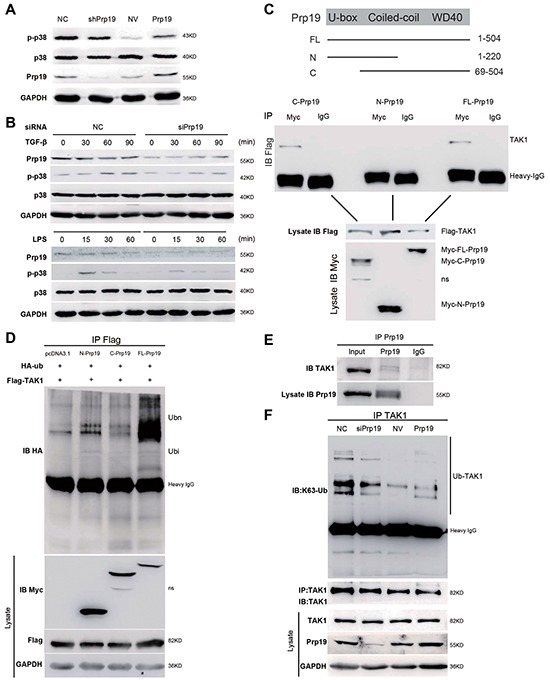
Prp19 facilitates k63-linked polyubiquitination on TAK1 to activate p38 MAPK in HCC cells **A.** Phosphorylation of p38 MAPK was detected in stable Huh7 cells mis-expressing Prp19. **B.** Huh7 cells transfected with indicated siRNAs were exposed to TGF-β (5 ng/ml) or LPS (1 μg/ml) for indicated time points, and followed by immunoblot. **C.** A schematic diagram of Prp19 domain depletion mutants (upper panel). Expression vectors of Myc-Prp19 and its domain-depletion mutants were cotransfected with Flag-TAK1 in 293T cells for 24h, and cell lysates were subjected to coimmunoprecipitation and western blot (lower panel). **D.** 293T cells were transfected with plasmids as indicated. Twenty-four hours after transfection, cell lysates were immunoprecipitated with anti-Flag. The immunoprecipitates were analysed by immunoblot with anti-HA. Whole cell lysates were analysed by immunoblot with indicated antibodies. **E.** Endogenous interaction between Prp19 and TAK1 was detected in Huh7 cells. **F.** Huh7 cells were transiently transfected with indicated siRNAs or plasmids for 48h, cell lysates were immunoprecipitated with TAK1 antibody, followed by western blot using k63 ubiquitin antibody. NC, negative control; NV, null vector. NS, nonspecific band.

In stable Huh7 transfectants, Prp19 expression was positively correlated with p-p38 level but not total p38 level (Figure 5A). Transient depletion of Prp19 in Huh7 cells also attenuated LPS- or transforming growing factor-β (TGF-β)-induced activation of p38/MAPK (Figure 5B). It is reported that k63-linked polyubiquitination of TGF-β-activated kinase 1 (TAK1) directly regulates TAK1 activity to activate p38/MAPK pathway [23]. In current study, *in vivo* binding between Prp19 and TAK1 was observed in 293T cells and Huh7 cells (Figure 5C and 5E, Supplementary Figure S8A), whereas depletion of WD40 domain abrogated it in 293T cells (Figure 5C). In contrast to null vector, full-length Prp19 efficiently facilitated polyubiquitination of TAK1, neither WD40 depletion mutant nor U-box depletion mutant could potently facilitate polyubiquitination of TAK1 (Figure 5D). Furthermore Prp19 directly bound with TAK1 *in vitro* in GST-pull-down analysis (Supplementary Figure S8B). In Huh7 cells, Prp19 facilitated k63-linked polyubiquitination but not k48-linked polyubiquitination of TAK1 (Figure 5F and Supplementary Figure S8C). The extent of k63-linked polyubiquitination of TAK1 was accordingly correlated with the phosphorylation of p38 in Huh7 cells, which partly contributed to the different phenotypes observed between NC-Huh7 cells and NV-Huh7 cells. The U-box domain containing E3 ligase activity rather than the WD40 domain was mainly responsible for Prp19-mediated activation of p38/MAPK (Supplementary Figure S8D). These data together suggest that Prp19 promotes p38/MAPK activation via binding with TAK1 to facilitate k63-linked polyubiquitination of TAK1 in HCC cells.

### Prp19 mediates invasion of HCC via p38 MAPK/Twist1 pathway

**Figure 6:**
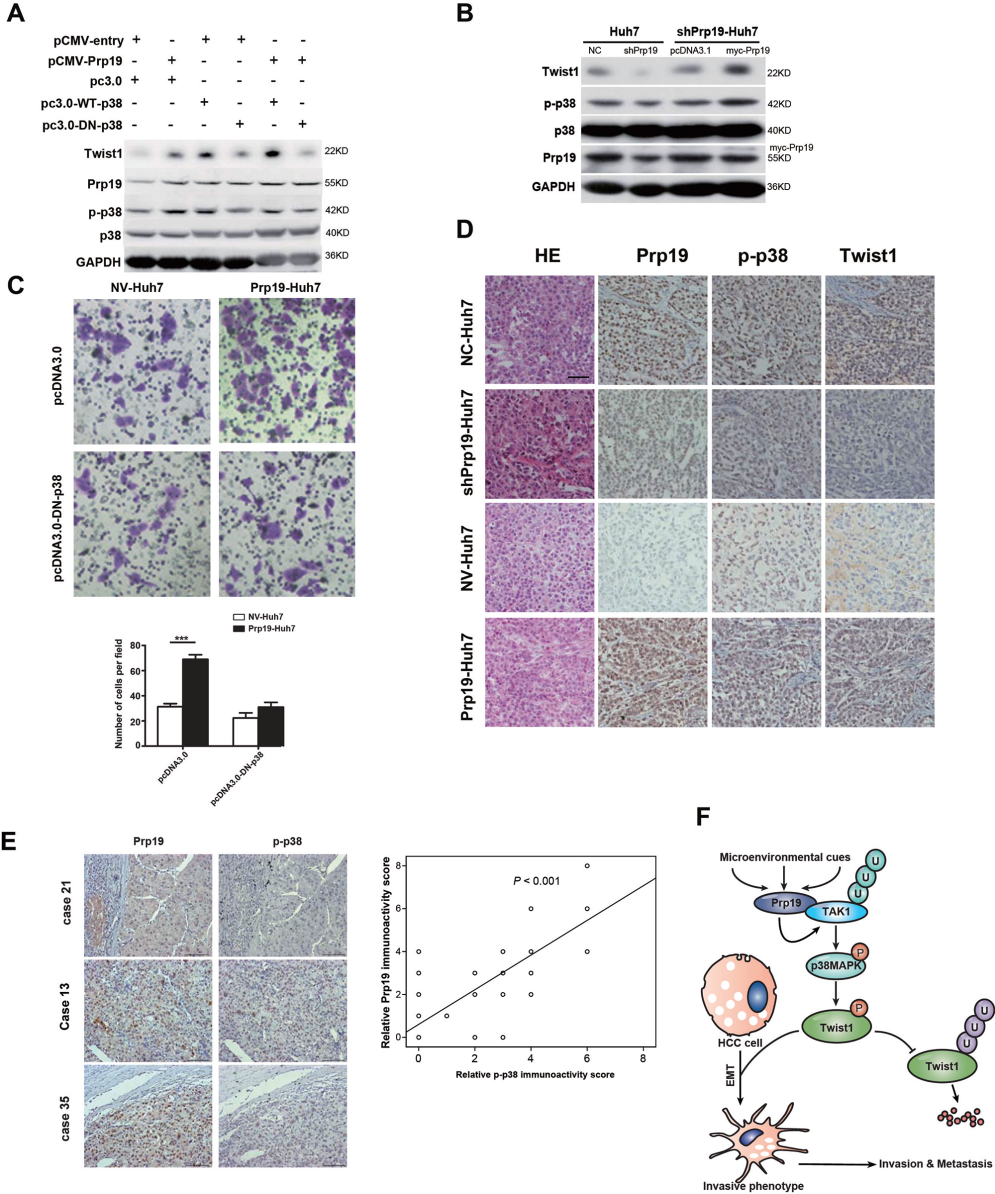
Prp19 mediates invasion of HCC via p38 MAPK/Twist1 pathway **A.** Huh7 cells were transfected with plasmids as indicated, cell lysates were analysed by immunoblot with indicated antibodies. **B.** shPrp19-Huh7 cells were transfected with myc-Prp19 and empty vector, followed by western blot. **C.** Prp19-Huh7 cells and NV-Huh7 cells were transfected with pcDNA3.0 or pcDNA3.0-DN-p38. The invasive capacities were analysed via Matrigel invasion chamber assay. **D.** Representative images of IHC staining of Prp19, p-p38 and Twist1 in the HCC tissues of orthotopic implantation model in nude mice (Original magnification ×200). **E.** Representative images of IHC staining with antibodies against Prp19, p-p38 in human HCC specimens (Original magnification ×200; left panel). Expression correlation of Prp19 and p-p38 was analysed in 80 HCC patients using IHC (right panel). **F.** Schematic presentation of the mechanism elucidating Prp19-mediated HCC invasion. NC, negative control; NV, null vector.

In Huh7 cells, Prp19-mediated increase of Twist1 was mostly blocked by dominant negative mutant (DN) or inhibitor of p38 (Figure 6A, Supplementary Figure S8E). Re-induction of Prp19 in part restored the activation of p38 MAPK as well as the expression of Twist1 in Prp19-deficient Huh7 cells (Figure 6B). In contrast to null vector, DN-p38 dramatically attenuated Prp19-elicited invasion of Huh7 cells. These results suggest that Prp19 promotes invasion of HCC cells via p38 MAPK/Twist1 pathway *in vitro* (Figure 6C).

To further verify the regulatory pathway *in vivo*, the association among Prp19, p-p38, and Twist1 was explored in tissues from orthotopic implantation model and human HCC specimens. In orthotopic implantation mice model, p38/MAPK phosphorylation and Twist1 expression were obviously increased in metastatic lesion of Prp19-Huh7 cells as compared to them in lesion of NV-Huh7 cells, and were markedly decreased in metastatic lesion of shPrp19-Huh7 cells as compared to them in lesion of NC-Huh7 cells (Figure 6D). IHC staining of human HCC specimens (n=80) revealed a close correlation between the expression levels of Prp19 and p-p38, which further supported Prp19-induced activation of p-p38 in human HCC (r = 0.765, Figure 6E). Overall, these data suggest that p38 MAPK/Twist1 pathway involves in Prp19-induced HCC invasion (Figure 6F).

## DISCUSSION

Ectopic activity of E3s is correlated with the pathology of cancer. Some E3s target tumor suppressors for ubiquitination-dependent degradation during the development of HCC [24, 25]. Wide survey of abnormal E3s in malignancies has indicated that Prp19 expression is higher in colon and larynx in contrast to their paracancerous tissues [26]. Herein we demonstrated that Prp19 expression in HCC was higher than that in their paired paratumor tissues, and its overexpression was positively correlated with vascular invasion, absent tumor capsule as well as poor prognosis. The pro-invasive role of Prp19 in HCC was validated both *in vitro* and *in vivo*. Overexpression of Prp19 observed in our study maybe attributed to aberrant UCH37 activity in HCC, which inhibits proteasome-mediated degradation of Prp19 [13].

To explore potential mechanisms, morphological alterations in HCC cells mis-expressing Prp19 arouse our interests. Based on the findings from cell lines and human tissues, we found that Prp19 promoted EMT of HCC. Since EMT often takes place at the edge of hepatic tumor [27], tumor cells with abnormal abundance of Prp19 was also observed both at the edge and matrix of HCC tissues in our study, which further supported the involvement of Prp19 during EMT (Supplementary Figure S8).

As a potent inducer of EMT, strictly control of cellular Twist1 levels could prevent tumor cells from acquiring invasive capacities [9]. In our study, Prp19-induced EMT in HCC cells was mainly dependent on Twist1. Opposed to the traditional role of E3s in targeting substrates for ubiquitin/proteasome-dependent degradation, Prp19 increased Twist1 stability in HCC cells. Phosphorylation of Ser 68 within Twist1 induced by p38/MAPK pathway has been characterized to be critical for its stability in breast cancer [21]. Here Ser68 mutant of Twist1 obviously attenuated Prp19-induced up-regulation of Twist1 in HCC cell, suggesting the involvement of Prp19 in the phosphorylation of Ser 68 within Twist1. Lately, activated p38/MAPK has been identified to promote EMT via up-regulating Twist1 or Snail expression in different types of tumor [21, 28]. In consistent with above studies, we found that inhibiting p38/MAPK activity repressed Twist1 expression in HCC cells.

TGF-β signaling pathway effectively activates p38/ MAPK and has a predominant role in the initiation of EMT [29]. Although UCH37 facilitates TGF-β signaling pathway, it is not required for TGF-β-dependent EMT [30, 31]. Our study showed that Prp19, as a potential effecter of UCH37, not only facilitated p38/MAPK activation but also induced EMT in HCC. It is reported that both LPS- and TGF-β-mediated p38/MAPK signaling are induced by TAK1 [32, 33], which is directly activated by k63-linked polyubiquitination [23]. The functions of Prp19 in pre-mRNA splicing and DNA damage response are dependent on its non-proteolytic E3 activity [34, 35], which is also required in activation of protein kinases [36]. We next identified that Prp19 directly bound with TAK1 and promoted k63-linked polyubiquitination on TAK1 in HCC cells. Considering both U-box domain depletion mutant and WD-40 domain depletion mutant evidently abolished polyubiquitination of TAK1, we proposed the synergic function of WD40 domain and U-box domain in Prp19-induced polyubiquitination of TAK1. However exact mechanism behind this program needs further exploration. Moreover, given the versatile roles of p38/MAPK pathway in tumor progression, efforts are needed to investigate whether Prp19-mediated invasion of HCC is dependent on other functions of p38/MAPK pathway.

In conclusion, our work demonstrates that Prp19 acts as an oncogene, and its overexpression promotes invasion by inducing EMT via, at least partially, the p38 MAPK/Twist1-dependent pathway in HCC. Since p38 blockade significantly overcomes therapy resistance of sorafenib in HCC mouse model [37], our work here provides possibilities that targeting Prp19 would be a promising therapeutic modality alone or together with other treatments for HCC.

## MATERIALS AND METHODS

### Patients and samples

All 169 HCC samples were randomly retrieved from patients who underwent curative resection and verified by postoperational histopathology at Liver Cancer Institute, Zhongshan Hospital of Fudan University from October 1, 2006 to December 31, 2008. The clinicopathologic characteristics of 169 patients were summarized in Supporting Table S1. Overall survival (OS) was defined as from time of surgery to last follow-up or time of death. The 13 normal liver tissues were obtained from patients with benign liver disease.

The protocol was approved by the Institutional Ethics Committee of Zhongshan Hospital of Fudan University (shanghai, China), and written informed consent was obtained from all study participants.

### Cell lines, shRNA lentivirus particles, siRNAs, plasmids, reagents

Human L02, 293T and Huh7, SMMC-7721, HepG2, SK-Hep1, MHCC-97H, MHCC-97L, and Hep3B cells were obtained from the Cell Bank of the Chinese Academy of Sciences (Shanghai, China) where they were characterized by mycoplasma detection, DNA-Fingerprinting, isozyme detection and cell vitality detection. All cell lines except L02 were cultured in DMEM with 10% fetal bovine serum (FBS). L02 was cultured in RPMI 1640 with 20% FBS. All these cells were cultured in a humidified incubator at 37 μC in the presence of 5 % CO_2_.

shRNA lentivirus particles containing three specific sequences against human Prp19 and its negative control shRNA (61415-V, 108080, Santa cruz) were used to transfect HCC cell lines together with 5 μg/mL polybrene (SC134220, Santa cruz) according to the manufacturer's protocol. SiRNAs against human Prp19, Twist1 and negative control siRNA were designed and synthesized by Genepharma (Shanghai, China). Human pCMV6-AC-Prp19 expression plasmid and vector control were purchased from OriGene (Rockville, USA). pReceiver-M13-Flag-Twist1 wild type and its Ser68A mutant were supplied by Genecopeia (Rockville, USA). Human Prp19 and its depletion mutants in pcDNA3.1/myc-His (−) vector and TAK1 in pRK7-Flag vector and GST-pGEX-4T-1 vector were generated according to the standard PCR protocol. All primers and siRNAs sequences were available on request. pRK5-HA-Ubiquitin, pcDNA3.0, pcDNA3.0-p38 wild type (WT) and pcDNA3.0-p38 dominant negative inhibitory mutant (DN) were gifts from other laboratories. Transfection of Plasmids and siRNAs were performed with Lipofectamine^®^2000 transfection reagent (Invitrogen, CA, USA) following the manufacturer's protocol. After transfection, stable cell lines were selected with puromycin or G418 (Sigma-Aldrich, St. Louis, USA). Antibodies recognizing Prp19 and Twist1 were purchased from Abcam Ltd (Cambridge, UK). Antibodies against p38, p-p38, ERK, p-ERK, JNK, p-JNK, TAK1, β-catenin, Snail, ZO-1, Zeb1 were from Cell Signaling Technology (Beverly, MA, USA). Detailed information about antibodies and chemical reagents were listed in Supplementary Table S6 and S7.

### Real-time PCR (qPCR) and western blot

Total RNA was exacted by TRIzol (Invitrogen, NY, USA). The first strand cDNA synthesis was carried out with AMV RNA PCR kit (TaKaRa, Dalian, China) according to the manufacturer's protocol. Subsequent real-time PCR was performed using a SYBR Green Premix Ex Taq (TaKaRa, Dalian, China) on ABI StepOne Plus system (Applied Biosystems, CA, USA). The primers of interested genes were available in Supplementary Table S5. Extracts of cell and HCC specimens were analyzed by western blot using ImageQuant LAS 4000 mini (GE Healthcare, New Jersey, USA).

### Immunofluorescence assay

Stable HCC cell lines mis-expressing Prp19 were incubated with antibody against N-cadherin and then incubated with anti-mouse IgG (Jackson ImmunoResearch Laboratories). The coverslips were counterstained with DAPI and imaged with a Nikon microscope with NIS Element F3.2 software (Nikon, Melville, NY, USA).

### Immunochemistry

Slides of paraffin-embedded primary HCC and adjacent paratumor tissues were used for immunochemistry and stained with antibodies against p-p38, Prp19 and Twist1, E-cadherin and N-cadherin. The exact procedure and the semi-quantitative method were reported elsewhere [15]. Twenty-nine samples from 169 HCC cases were scored as 0. The optimum cut-off value for immunoactivity score was defined by receiver operating curve analysis as described in Supplementary Table S3. Images were processed with a Nikon microscope with NIS Element F3.2 software (Nikon, Melville, NY, USA).

### *In vitro* cell behaviour assays

The mobility of HCC cells was assessed by wound-healing assay and Transwell insert assay. The invasive capacity of HCC cells was assessed by soft-agar colony formation assay and by Matrigel invasion chamber assay. The detailed information was as described elsewhere [16]. For Cell proliferation assay, shPrp19-Huh7, Prp19-Huh7 and their control cells (1.5 × 10^3^) were cultured in 96-well plate for various time periods. ATP activity was measured using Cell Counting Kit-8 (Beyotime, Nantong, China) with an All-in-One Microplate Reader (Bioteck, USA) according to the manufacturer's protocol.

### GST pull-down and immunoprecipitation analysis

Briefly, GST and GST-TAK1 were amplified in *E coli* BL21 and purified, followed by incubation with His6-Prp19 expressed by 293T cells respectively. Then the products were analyzed by immunoprecipitation analysis. Huh7 and 293T cells under corresponding treatments were harvested in immunoprecipitation lysis buffer supplemented with a complete protease inhibitor cocktail and a phosphatase inhibitor cocktail (Roche, Mannheim, Germany). The cell lysates were immunoprecipitated with corresponding antibodies and detected by western blot.

### Mice

Animal care and experimental protocols were conducted in accordance with guidelines established by the Experimental Animal Care Commission of Shanghai Medical School of Fudan University (Shanghai, China). BALB/C nude mice (4-5 weeks old, male) were purchased from Shanghai Institute of Material Medicine of Chinese Academy of Science, and raised in specific pathogen-free conditions. For in situ transplantation model, 28 nude mice were randomized into four groups. 10^7^ stable HCC cells mis-expressing Prp19 in 200 μL PBS were inoculated subcutaneously into the left armpit of nude mice (n = 2). 4 weeks later, xenograft tumors were removed and transplanted into the left lateral lobe of liver in remaining nude mice (n=5). Four weeks later, metastatic lesions on the surface of liver were detected. For the tail vein metastasis model, 80 nude mice were randomized into four groups. 2×10^6^ stable HCC cells mis-expressing Prp19 in 100 μL PBS were injected into the tail vein of nude mice. Thirteen mice in each group were sacrificed at 4 weeks after inoculation and consecutive sections of the whole lung were subjected to hematoxylin and eosin staining. All of the metastatic foci in lung were calculated microscopically to evaluate pulmonary metastasis. The remaining mice were observed for survival analysis.

### Statistical analysis

Statistical analysis was carried out using SPSS version 19.0 (Chicago, IL, USA). Data were expressed as means or standard errors of the mean from three independent studies. Student *t* test, the Mann-Whitney *U* test or one way analysis of variance was applied to compare significant differences between different groups. Kaplan-Meier and log-rank test were used to assess patient survival between subgroups. Statistical significance was set at *P* < 0.05.

## SUPPLEMENTARY FIGURES AND TABLES


