# Supplementary material for: Prp19 facilitates invasion of hepatocellular carcinoma via p38 mitogen-activated protein kinase/Twist1 pathway
Source: Oncotarget. 2016 Mar 3;7(16):21939–51. doi: 10.18632/oncotarget.7877 (PMC5008335; doi:10.18632/oncotarget.7877)
Supplement: Supplementary file 1 [file oncotarget-07-21939-s001.pdf]

SUPPLEMENTARY FIGURES AND TABLES

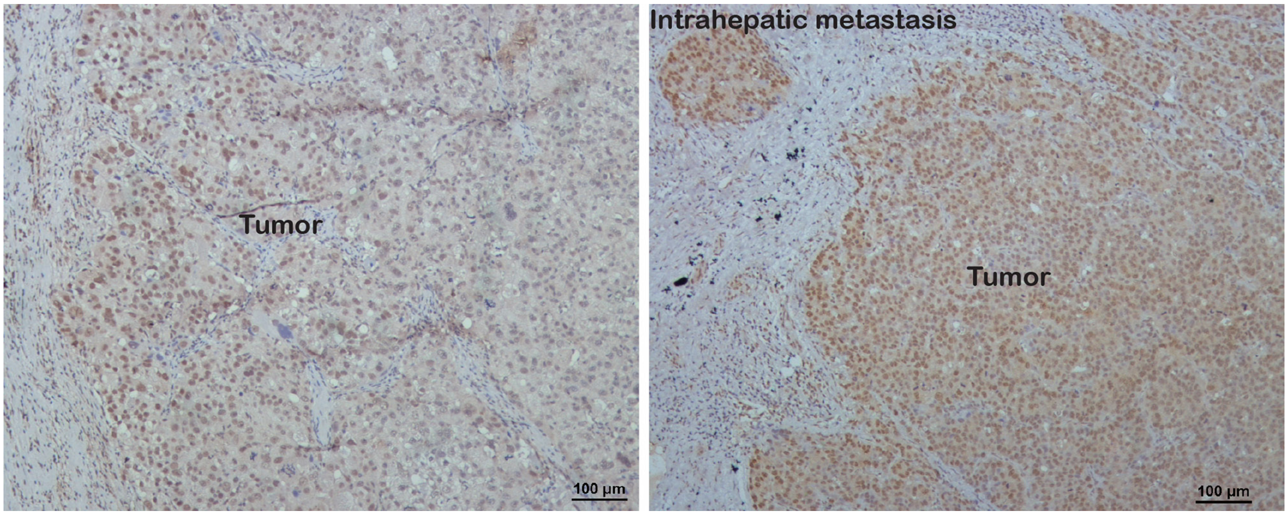

Supplementary Figure S1: Tumor cells at the edge (Left panel) and adjacent metastatic lesion (Right panel) of HCC tissues displayed enhanced Prp19 expression (Original magnification ×200).

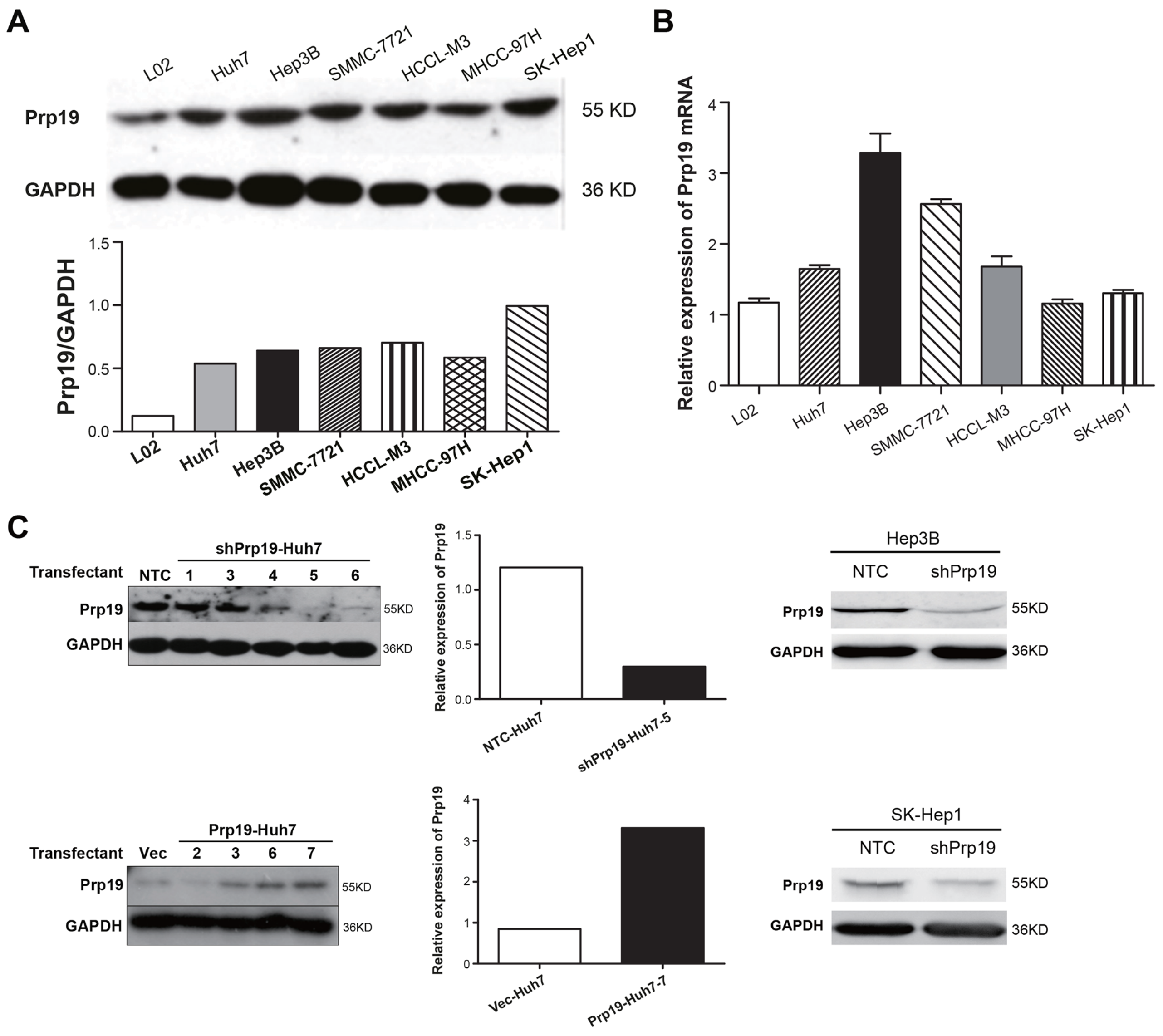

Supplementary Figure S2: A. The protein expression of Prp19 in normal hepatocyte L02 and six HCC cell lines. Prp19/GAPDH ratios were presented in bar chart. **B.** Relative mRNA level of Prp19 in normal hepatocyte L02 and six HCC cell lines. **C.** Identification of stable transfectants of Huh7, Hep3B and SK-Hep1 cells mis-expressing Prp19.

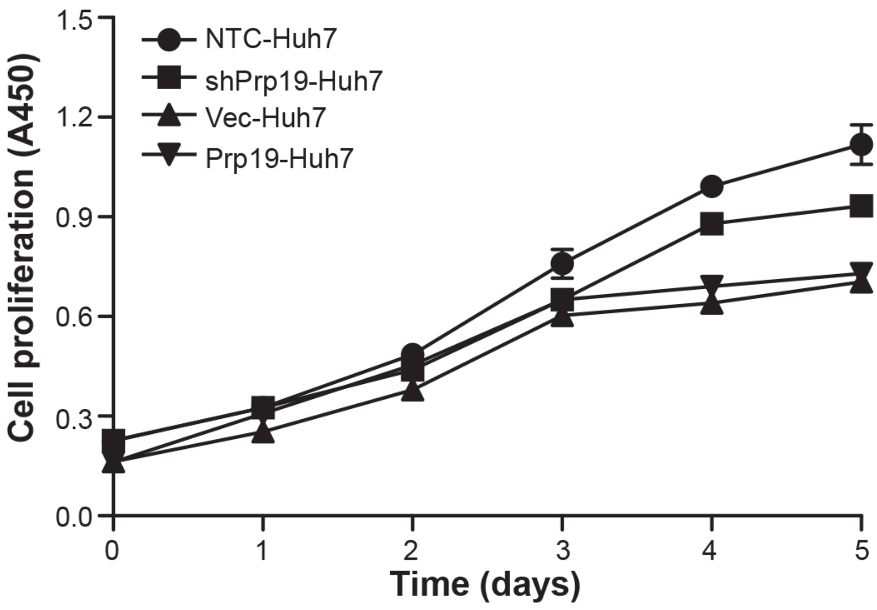

Supplementary Figure S3: Stable Huh7 cells mis-expressing Prp19 were seeded into a 96-well plate, and Cell Counting Kit 8 assay was performed at indicated time points determining the effect of Prp19 on HCC cells growth *in vitro*.

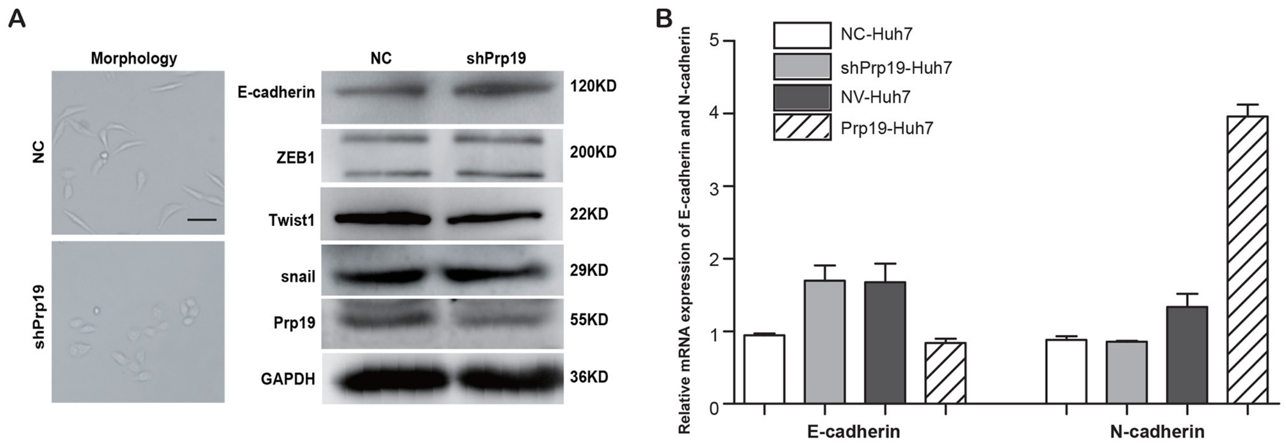

Supplementary Figure S4: A. Morphology (left panel) and expression of EMT markers (right panel) in stable transfectants of SMMC-7721 mis-expressing Prp19 (black scale bar: 100μm). **B.** Relative mRNA expression of E-cadherin and N-cadherin in stable Huh7 cells mis-expressing Prp19.

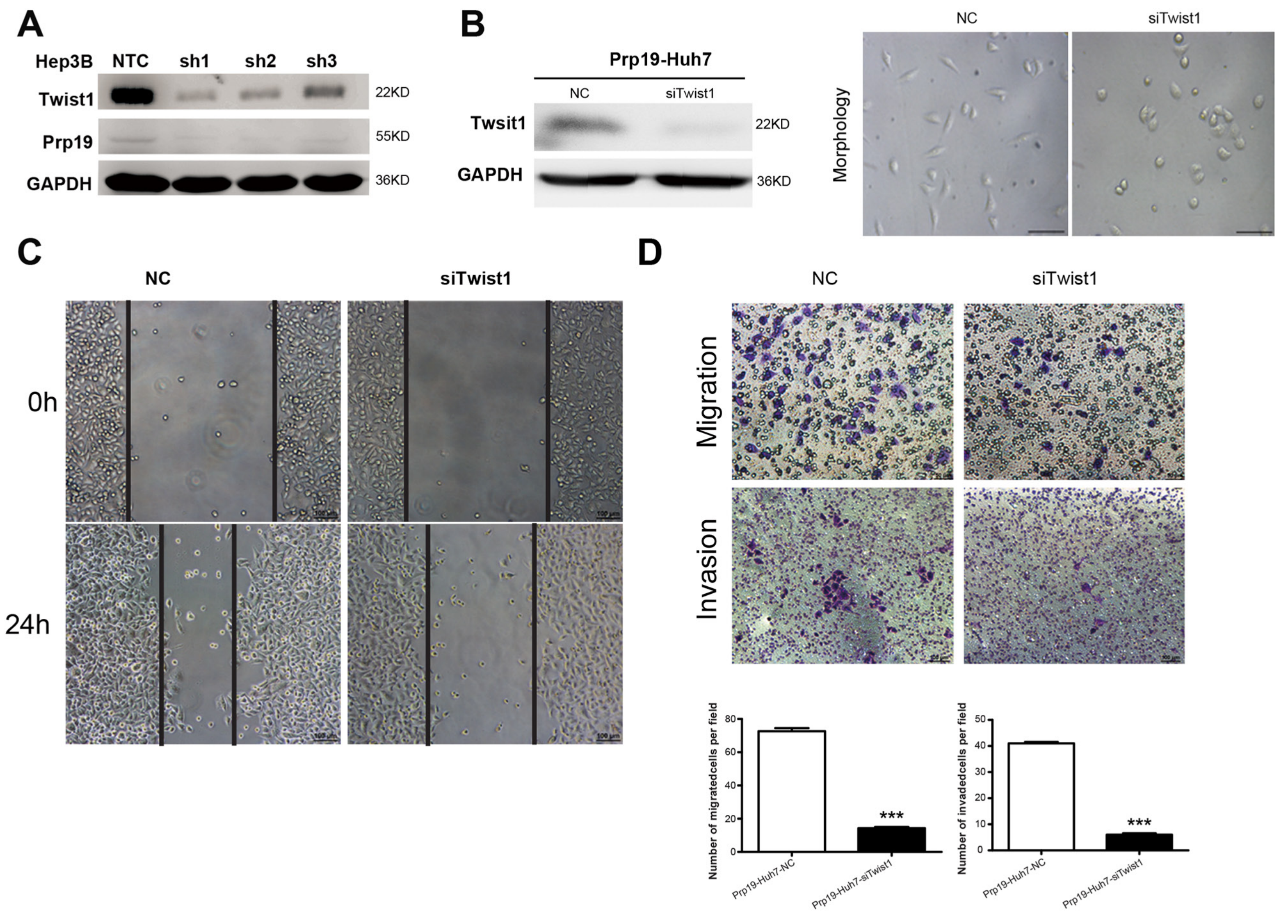

Supplementary Figure S5: A. Prp19 and Twist1 expression were analyzed by immunoblot in three stable transfectants of Hep3B cells mis-expressing Prp19. **B.** Prp19-Huh7 cells were transfected with indicated siRNAs for 72h, and then morphology was presented. **C, D.** Prp19-Huh7 cells were transfected with indicated siRNAs for 48h hours, then subjected to wound-healing assay, transwell assay or Matrigel invasion chamber assay. ****P* < *0.001*.

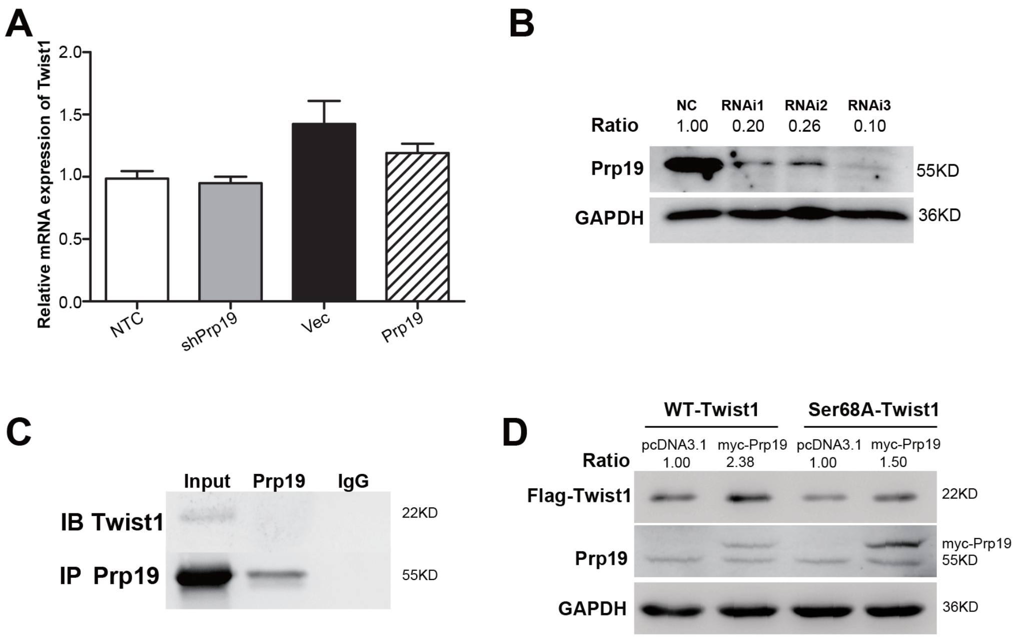

Supplementary Figure S6: A. Relative mRNA level of Twist1 in stable Huh7 cells mis-expressing Prp19. **B.** Endogenous interaction between Prp19 and Twist1 was assessed in Huh7 cells. **C.** Huh7 cells were transfected with indicated siRNAs for 72h, followed by western blot. **D.** SK-Hep1 cells were transfected with indicated plasmids, followed by western blot. Densitometric values of Flag were detected and presented.

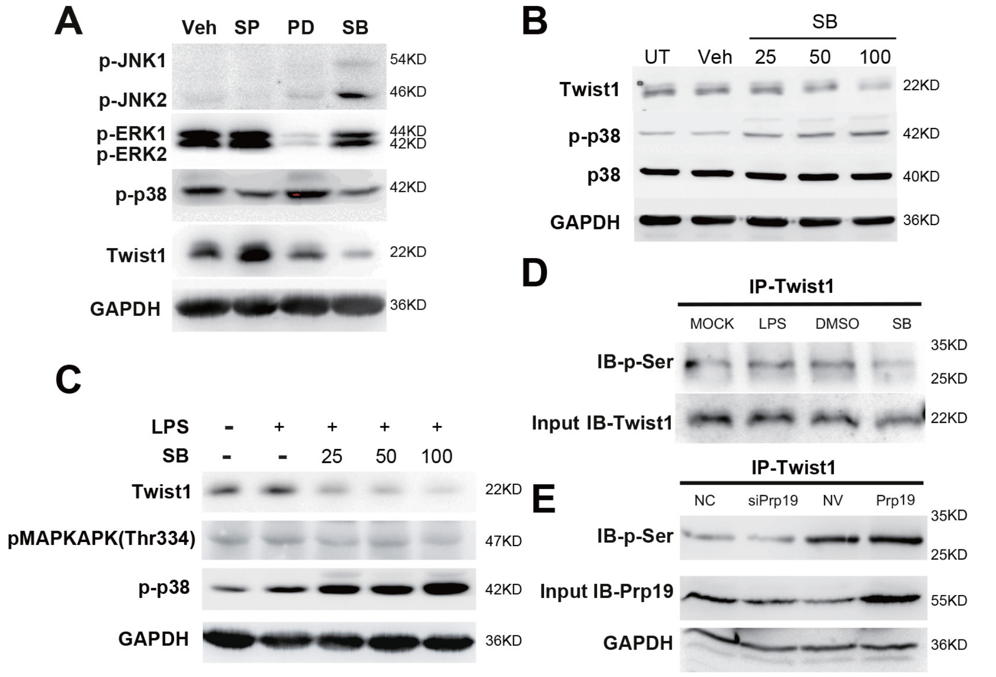

Supplementary Figure S7: A. Huh7 cells were transfected with indicated siRNAs or plasmids for 48h. Total ser phosphorylation within Twist1 was assessed by immunoprecipitation. **B.** Huh7 cells were incubated with 50μM SP600125, 25μM PD980059 or 100 μM SB203580 for 2h, and subjected to western blot. **C.** Huh7 cells were treated with SB203580 at different concentrations for 2h, followed by Western blot. **D.** Huh7 cells were treated with 1 μg/mL LPS for 0.5h, and following treated with SB203580 at different concentrations for another 2h. p-MAPKAPK2 was used as an indicator of p38 MAPK activity. **E.** Huh7 cells were treated with 100 μM SB203580 or 1μg/mL LPS for 2h. Total ser phosphorylation within Twist1 was assessed by immunoprecipitation.

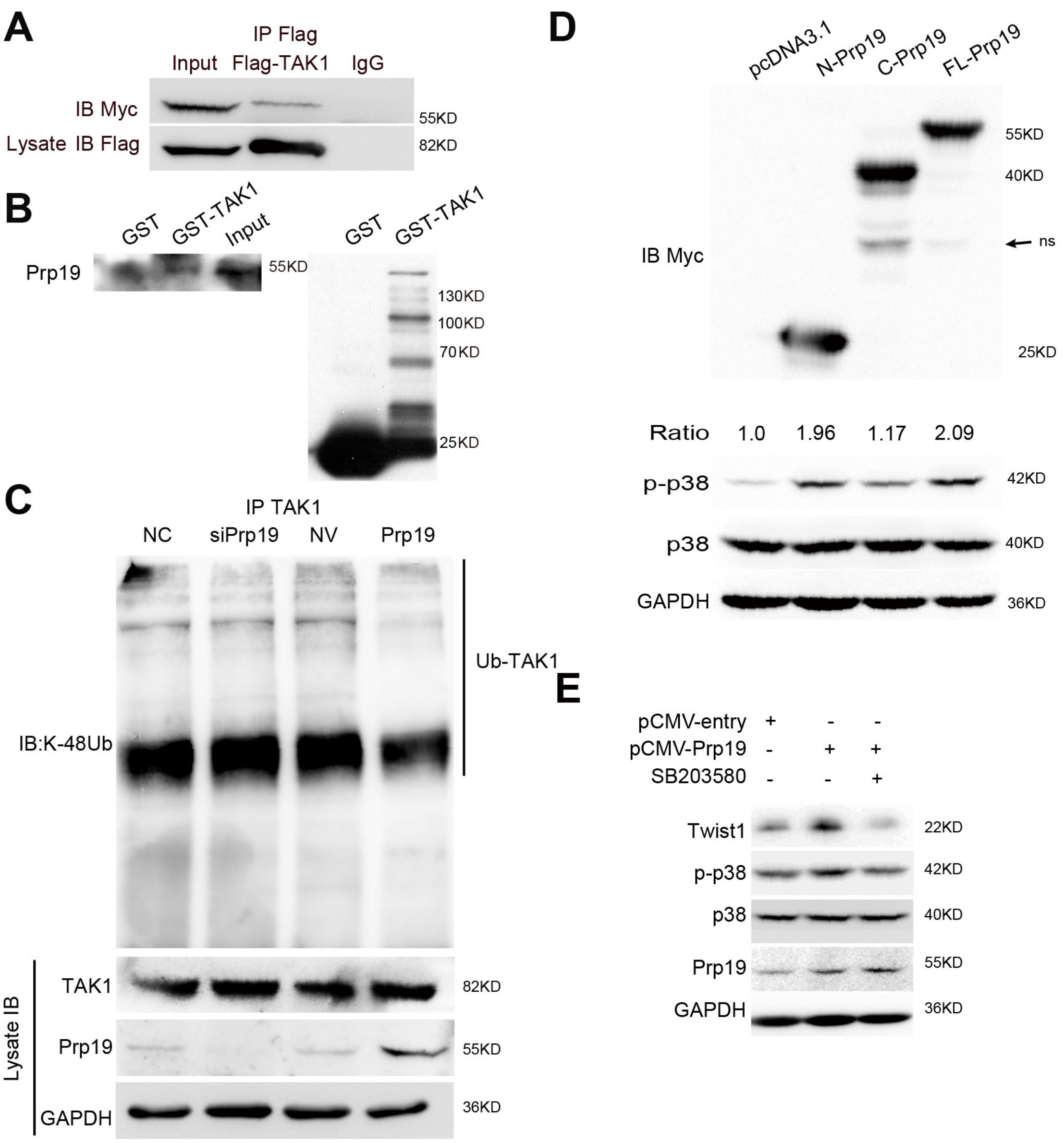

Supplementary Figure S8: A. 293T cells were transfected with Myc-Prp19 and Flag-TAK1. Twenty-four hours after transfection, cell lysates were immunoprecipitated with Flag antibody. The immunoprecipitates were analyzed by immunoblots with anti-Myc. **B.** The binding of His6-Prp19 and GST-TAK1 was analyzed in vitro. **C.** Huh7 cells were transfected with the indicated siRNAs or plasmids for 48h. Before harvest, cells were treated with 20 μM MG132 for another 6h. Cell lysates were immunoprecipitated with TAK1 antibody, followed by western blot using k-48 ubiquitin antibody. **D.** 293T cells were transfected with expression vector of Myc-Prp19 or its domain-depletion mutants, followed by western blot. Densitometric values of p-p38 were presented after normalization. **E.** Huh7 cells were transfected with indicated plasmids, and following treated with SB203580 100μM for 2h. Cell lysates was analyzed by western blot.

Supplementary Table S1: Summary of Clinicopathologic Variables

Characteristics	Number of Patients
Patients	169
Sex	
Male	143
Female	26
Age (years)	24-79, median = 53
HBV	
Positive	151
Negative	18
Tumor size (cm)	1-19, median = 5
Vascular invasion	
Yes	96
No	83
Tumor capsule	
Present	120
Absent	49
intrahepatic metastasis	
Yes	61
No	108
Distant metastasis	
Present	5
Absent	164
TNM stage	
I- II	98
III-IV	71
Time of follow-up (months)	0.5-74.1, median = 39.2

Abbreviations: HBV, hepatitis B virus surface antigen positive; TNM, tumor-nodes-metastasis, based on the American Joint Committee on Cancer/International Union Against Cancer staging system (7th edition, 2009).

Supplementary Table S2: Immunoactivity Score of Intratumoral/Peritumoral Prp19

Prp19 staining	Score		t	*P* value
	Mean ± SD	Range		
Intratumor	2.46±1.607	0–8	6.811	0.000**
Peritumor	1.70±1.229	0–6		

NOTE: **Paired Wilcoxon signed-rank test.

Supplementary Table S3: ROC Analysis of Intratumoral Prp19 immunoactivity Score

Immunoactivity Score	Area	Standard Error	Asymptopic Significance	95% CI
2	0.550	0.044	0.263	0.463 - 0.637
3	0.561	0.044	0.172	0.474 - 0.648
**4**	**0.570**	**0.044**	**0.116**	**0.483 - 0.657**
6	0.484	0.045	0.727	0.397 - 0.572

Abbreviations: ROC, receiver operating curve. CI, confidence interval

Supplementary Table S4: Relationship between Prp19 Expression and Clinicopathologic Features of HCC Patients

Features		Prp19 expression		*P* Value
		Low	High	
Sex	Male	107	36	0.628
	Female	18	8	
Age	≤55	71	31	0.151
	>55	54	13	
HBV	Positive	111	40	1.000
	Negative	14	4	
Tumor size (cm)	≤5	71	21	0.379
	>5	54	23	
Capsular invasion	Yes	53	30	**0.005**
	No	72	14	
Tumor capsule	Present	97	23	**0.002**
	Absent	28	23	
Intrahepatic metastasis	Yes	41	20	0.147
	No	84	24	
Distant metastasis	Present	4	2	0.539
	Absent	124	39	
TNM stage	I- II	76	22	0.220
	III-IV	49	22	

Note: HCC patients were divided into Prp19 'High' group (immunoactivity score≥4) and 'Low' group (immunoactivity score<4). The patient and disease profiles in each group were compared.
Abbreviations: HBV Positive, hepatitis B virus surface antigen positive; TNM, tumor-nodes-metastasis; Differences among variables were assessed by χ^2 analysis.

Supplementary Table S5: Primers Sequence for qPCR and Expression Plasmid Construct

Primer	Sequence (5' to 3')
Prp19 forward primer	GTGCC AAGTT CCCAA CCAAG TGTT
Prp19 reverse primer	AGCAC AGTGG CTTTG TCTTG AAGC
Myc-Prp19 forward primer	CCGCT CGAGA TGTCC CTAAT CTGCT CCATC TCT
Myc-Prp19 reverse primer	CCGGA ATTCTCAGGCTGTAGAACTTGAG
Myc-WD-Prp19 forward primer	CCGCT CGAGA TGTCC CTAAT CTGCT CCATC TCT
Myc-WD-Prp19 reverse primer	CCGGA ATTCT GCTGG CACTG TGCAA CCCC
Myc-UD-Prp19 forward Primer	CCGCT CGAGA TGACC AGCAT CCCG
Myc-UD-Prp19 reverse Primer	CCGGA ATTCT CAGGC TGTAG AACTT GAG
Flag-TAK1 forward primer	CGCGG ATCCA TGTCT ACAGC CTCTG CC
Flag-TAK1 reverse primer	CCGGA ATTCT CATGA AGTGC CTTGT CG
Twist1 forward primer	GGGAG TCCGC AGTCT TACGA
Twist1 reverse primer	AGACC GAGAA GGCGT AGCTG
E-cadherin forward primer	TACAC TGCCC AGGAG CCAGA
E-cadherin reverse primer	TGGCA CCAGT GTCCG GATTA
N-cadherin forward primer	CACGC CGAGC CCCAG TAT
N-cadherin reverse primer	GCCCC CAGTC GTTCA GGTAA
GAPDH forward primer	TCGAC AGTCA GCCGC ATCTT CTTT
GAPDH reverse primer	GCCCA ATACG ACCAA ATCCG TTGA

Supplementary Table S6: List of siRNAs Sequence Used for Transfection

siRNAs	Sequence (5' to 3')
siRNA1 Prp19 sense	GGCUC AUCGA GAAGU ACAUT T
siRNA1 Prp19 antisense	AUGUA CUUCU CGAUG AGCCT T
siRNA2 Prp19 sense	GCCAC UAUCA GGAUU UGGUT T
siRNA2 Prp19 antisense	ACCAA AUCCU GAUAG UGGCT T
siRNA3 Prp19 sense	GCCAA GUUCA UCGCU UCAAT T
siRNA3 Prp19 antisense	UUGAA GCGAU GAACU UGGCT T
siRNA Twist1 sense	UUGAG GGUCU GAAUC UUGCU CAGCU
siRNA Twist1 antisense	AACUG AGCAA GAUUC AGACC CUCAA
siRNA Negative control sense	UUCUC CGAAC GUGUC ACGUT T
siRNA Negative control antisense	ACGUG ACACG UUCGG AGAAT T

Supplementary Table S7: List of the Primary Antibodies Used for Immunoblotting, Immunohistochemistry and Immunoprecipitation Analysis

Proteins	Antibody	Epitope mapping	Manufacturers
Prp19	Mouse monoclonal	N-terminus	Santa Cruz Biotechnology, Santa Cruz, CA
Prp19	Rabbit Polyclonal	C-terminus	Abcam Ltd, Cambridge, UK
Epithelial-Mesenchymal Transition(EMT) Antibody Sample Kit	Rabbit		Cell Signaling Technology, Beverly, MA, USA
N-cadherin	Mouse monoclonal	N-terminus	Santa Cruz Biotechnology, Santa Cruz, CA
E-cadherin	Mouse monaclonal		Santa Cruz Biotechnology, Santa Cruz, CA
Twist (Twist2C1a)	Mouse monoclonal	C-terminus	Santa Cruz Biotechnology, Santa Cruz, CA
Twist (ab49254)	Rabbit polyclonal	C-terminus	Abcam Ltd, New territories, HK
p-MAPKAPK-2	Rabbit monoclonal	Threonine 334	Santa Cruz Biotechnology, Santa Cruz, CA
Ubiquitin	Mouse monoclonal	Full length	Cell Signaling Technology, Beverly, MA, USA
K-63 linkage specific polyubiquitin	Rabbit monoclonal		Cell Signaling Technology, Beverly, MA, USA
K-48 linkage specific polyubiquitin	Rabbit monoclonal		Cell Signaling Technology, Beverly, MA, USA
p38 MAPK	Rabbit monoclonal	C-terminus	Cell Signaling Technology, Beverly, MA, USA
TAK1	Rabbit monoclonal		Cell Signaling Technology, Beverly, MA, USA
Phospho-p38 MAPK	Rabbit monoclonal	Threonine180 Tyrosine182	Cell Signaling Technology, Beverly, MA, USA
p44/42 MAPK	Rabbit monoclonal	C-terminus	Cell Signaling Technology, Beverly, MA, USA
Phospho-p44/42 MAPK	Rabbit monoclonal	Threonine202 Tyrosine204	Cell Signaling Technology, Beverly, MA, USA
SAPK/JNK	Rabbit monoclonal	Full length	Cell Signaling Technology, Beverly, MA, USA
Phospho-SAPK/JNK	Rabbit monoclonal	Threonine183 Tyrosine185	Cell Signaling Technology, Beverly, MA, USA
Flag-Tag	Mouse monoclonal		Sigma-Aldrich, St. Louis, MO
Myc-Tag	Mouse monoclonal	C-terminus	Millipore, Billerica, MA
HA-Probe	Mouse monoclonal		Santa Cruz Biotechnology, Santa Cruz, CA
GAPDH	Mouse monoclonal	Full length	Santa Cruz Biotechnology, Santa Cruz, CA

Supplementary Table S8: List of Chemical Reagents Used for the Study

Chemical reagents	Application	Manufacturers
SB203580	Specific p38 MAPK inhibitor	Selleck, Houston, TX
PD980059	Selective ERK1/2 inhibitor	Selleck, Houston, TX
SP600125	JNK inhibitor	Selleck, Houston, TX
Lipopolysaccharide (LPS)	p38/MAPK Stimulant	Sigma-Aldrich, St. Louis, MO
MG132	Proteasome inhibitor	Merck KGaA, Darmstadt, Germany
Cycloheximide (CHX)	Protein synthetise inhibitor	Beyotime Instituted of Biotechnology, China
